# Omentin-1 and cardiovascular disease: a comprehensive review from molecular mechanisms to clinical translation

**DOI:** 10.3389/fendo.2026.1967354

**Published:** 2026-09-25

**Authors:** Yingcan Zhang, Wei Hao, Meiqi Luan, Juzheng Xiao, Yueyi Liang, Dongyu Zhan, Bo Yu, Li Yin, Fang Liu

**Affiliations:** 1 Department of Cardiology, The Second Affiliated Hospital of Harbin Medical University, Harbin, China; 2 The Key Laboratory of Myocardial Ischemia, Chinese Ministry of Education, Harbin, China; 3 State Key Laboratory of Frigid Zone Cardiovascular Diseases, Harbin Medical University, Harbin, China

**Keywords:** adipokines, atherosclerosis, biomarkers, cardiovascular diseases, clinical translation, Omentin-1

## Abstract

Omentin-1 is an adipokine predominantly secreted by visceral adipose tissue (VAT) and is abundant in human plasma. Circulating omentin-1 levels are significantly reduced in patients with obesity, type 2 diabetes mellitus (T2DM), hypertension, and coronary artery disease (CAD). However, the associations between circulating omentin-1 levels and cardiovascular risk and prognosis appear heterogeneous across populations and disease stages. Accumulating evidence indicates that omentin-1 exerts multiple cardioprotective effects. These effects include anti-atherosclerotic activity, improved insulin sensitivity, anti-inflammatory and antioxidant effects, enhanced endothelial function, and myocardial protection. Through these mechanisms, omentin-1 contributes to vascular and cardiometabolic homeostasis. This review summarizes the biological characteristics of omentin-1 and its protective roles and molecular mechanisms in CVD. It also highlights recent advances in clinical research. We further highlight emerging evidence that commonly used cardiometabolic drugs can modulate omentin-1 levels, supporting its potential as both a biomarker and a therapeutic target. A clearer understanding of its pleiotropic actions may facilitate the development of translational strategies for CVD prevention and treatment.

## Highlights

- This review covers molecular mechanisms and clinical correlations, and critically interprets the controversies in current research—including the bidirectional changes in omentin-1 levels in acute coronary syndrome (ACS) as well as population-dependent prognosis. It identifies research gaps, points out future research directions, and clarifies the clinical utility of omentin-1 to guide translational applications.

## 1 Introduction

Cardiovascular disease (CVD) remains a major global public health challenge. Its prevalence continues to rise, and it remains the leading cause of death in both urban and rural populations in China (1). Despite advancements in diagnosis and treatment that have reduced in-hospital mortality, the burden of CVD remains substantial. Therefore, improving early diagnosis, optimizing treatment strategies, and enhancing prognostic management are critical for reducing the burden of CVD.

In recent years, the adipokine omentin-1 has attracted increasing attention for its potential role in early screening, diagnosis, and prognostic assessment of CVD. Unlike adipokines such as leptin and resistin, which are typically elevated in obesity and contribute to inflammation and insulin resistance, omentin-1 levels are reduced in patients with obesity, type 2 diabetes mellitus (T2DM), metabolic syndrome (MetS), and CVD (2–4).

Studies indicate that reduced omentin-1 levels are independently associated with increased CVD risk, suggesting a key protective role in metabolic and cardiovascular homeostasis. Omentin-1 has been shown to exert multiple protective effects, including anti-atherosclerotic activity, improved insulin sensitivity, anti-inflammatory actions, amelioration of endothelial dysfunction, and direct cardioprotective effects (5–7). Together, these findings highlight a close association between omentin-1 and CVD pathogenesis and support its potential role as both a biomarker for cardiovascular risk stratification and a therapeutic target.

Therefore, this review summarizes recent advances in omentin-1 research, including its biological characteristics, cardioprotective mechanisms, clinical relevance, and therapeutic potential, to provide new insights into CVD prevention and management.

### 1.1 Literature search strategy

This article was designed as a narrative review rather than a systematic review or meta-analysis. Relevant literature was identified through searches of PubMed/MEDLINE, Embase, Web of Science, and the China National Knowledge Infrastructure (CNKI), supplemented by screening the reference lists of relevant publications. The literature search covered studies available through July 2026. Search terms included combinations of “omentin-1”, “omentin”, “intelectin-1”, “cardiovascular disease”, “coronary artery disease”, “atherosclerosis”, “acute coronary syndrome”, “myocardial infarction”, “heart failure”, “hypertension”, “endothelial dysfunction”, “vascular remodeling”, “inflammation”, “oxidative stress”, “insulin resistance”, “adipokine”, “biomarker”, and “therapy”.

Peer-reviewed studies relevant to the biological characteristics, cardiovascular mechanisms, clinical associations, prognostic implications, and potential therapeutic modulation of omentin-1 were prioritized. Evidence from *in vitro* studies, animal models, observational and prospective human studies, and pharmacological investigations was considered to provide an integrated mechanistic-to-clinical perspective. Relevant reviews were additionally consulted for background information and identification of pertinent primary studies. Representative publications were selected according to their relevance to the scope of this review, methodological robustness, and contribution to the mechanistic or clinical questions discussed. Because this is a narrative review, the literature selection was intended to provide a balanced and representative synthesis of the available evidence rather than an exhaustive systematic evidence analysis.

## 2 Biological characteristics of Omentin-1

Adipose tissue is not merely an energy storage organ but also secretes various adipokines (e.g., adiponectin, leptin, resistin, omentin-1) involved in the regulation of metabolic and cardiovascular homeostasis (8). Omentin is a fat-specific secretory factor present in human circulation. It exists in two homologous isoforms, omentin-1 and omentin-2, of which omentin-1 is the predominant circulating form. Omentin-1, also known as intelectin-1 (9), is an anti-inflammatory adipocytokine composed of 313 amino acids. It belongs to the family of secretory glycoproteins, with a molecular weight of approximately 34 kDa. The omentin-1 gene is located on chromosome 1q22–q23, a region linked to genetic susceptibility to T2DM (10), which is a major risk factor for CVD. This suggests that omentin-1 may be involved in CVD pathogenesis through genetic mechanisms. The mature omentin-1 glycoprotein consists of 295 amino acids and is secreted into the circulatory system to exert its physiological functions (5, 10).

The expression of the omentin-1 gene is significantly higher in visceral adipose tissue (VAT) than in subcutaneous adipose tissue (SAT) (11). In rodent models, epididymal adipose tissue (as a VAT surrogate) is the primary source of omentin-1 (9). Research shows that omentin-1 mRNA expression is much higher in non-adipocyte cells of adipose tissue, such as stromal vascular cells, than in adipocytes. Adipocytes account for only about 9% of total omentin-1 mRNA expression, indicating that they are not the main source of omentin-1 secretion; rather, stromal cells within adipose tissue are the primary source (9, 12). In summary, omentin-1 is expressed in tissues such as the greater omentum, epicardial adipose tissue (EAT), and perivascular adipose tissue, but is primarily produced by stromal vascular cells in visceral fat depots (especially EAT and omental fat) and is abundant in plasma (13).

## 3 Cardiovascular protective mechanisms of Omentin-1

The cardiovascular protective effects of omentin-1 are not mediated through a single pathway. Instead, they involve multiple coordinated mechanisms and targets that contribute to the development and progression of CVD.

### 3.1 Anti-atherosclerotic axis

Atherosclerosis is a complex pathological process involving abnormal lipid accumulation, vascular smooth muscle cell activation and vascular remodeling, metabolic dysfunction, chronic vascular inflammation, and oxidative stress. Vascular remodeling is one of its core pathological features. During disease progression, vascular smooth muscle cells (VSMCs) migrate from the media to the intima, proliferate abnormally, and contribute to extracellular-matrix remodeling, thereby promoting intimal thickening, vascular stiffening, and plaque progression (14, 15). As an adipokine with experimentally demonstrated cardioprotective properties, omentin-1 may counteract atherogenesis through coordinated regulation of VSMC activity and vascular remodeling (14, 15), lipid handling and cholesterol homeostasis (14, 16, 17), insulin signaling (18), and inflammatory and oxidative pathways (19–22).

In addition to adipokine-mediated regulation, vascular responses to oxidized low-density lipoprotein (oxLDL) may also involve noncoding RNA networks. Bao et al. demonstrated that the LINC00657/miR-590-3p regulatory axis contributes to low-concentration oxLDL-induced angiogenesis, providing complementary evidence that neovascularization and plaque remodeling during atherosclerosis are regulated by multiple molecular mechanisms (23). This finding broadens the mechanistic context of lipid-associated vascular injury; however, it should not be interpreted as evidence that omentin-1 directly regulates the LINC00657/miR-590-3p pathway.

#### 3.1.1 Inhibition of pathological activation of VSMCs and vascular remodeling

The regulatory effect of omentin-1 on VSMCs is central to its inhibition of vascular remodeling. Omentin-1 has been shown to inhibit angiotensin II (Ang II)-induced migration of human aortic smooth muscle cells and to antagonize PDGF-BB-induced proliferation (14). These effects suppress the pathological activation of VSMCs at both the migration and proliferation stages. Studies in mouse models further confirm that omentin-1, dependent on the AMP-activated protein kinase (AMPK) activation pathway, effectively inhibits VSMCs abnormal proliferation and neointimal hyperplasia after arterial injury (15, 24). During vascular remodeling, omentin-1 bidirectionally regulates matrix metabolism: it downregulates pro-fibrotic components (e.g., collagen-I, collagen-III) to attenuate wall fibrosis and stiffness, while upregulating key elastic components (e.g., elastin, fibronectin), thereby preserving vascular integrity and function (14).

#### 3.1.2 Improving abnormal lipid metabolism

Omentin-1-mediated regulation of lipid metabolism represents a key mechanism by which it inhibits atherosclerosis progression. This effect is achieved through the synergy between modulating local cholesterol metabolism in the vessel wall and maintaining systemic lipoprotein homeostasis.

Foam cells, major constituents of the early lipid core in atherosclerosis, form extensively during the initial phase of pathological lipid deposition in the arterial wall. Omentin-1 can inhibit oxLDL-induced foam cell formation by downregulating scavenger receptors (CD36, SR-A) and ACAT-1, and upregulating NCEH and ATP-binding cassette transporter A1 (ABCA1), thereby reducing cholesterol ester accumulation in macrophages (14, 24).

A study indicates that circulating omentin-1 levels positively correlate with anti-atherogenic high-density lipoprotein (HDL) and negatively correlate with pro-atherogenic very-low-density lipoprotein (VLDL) (16). Research in ApoE^−^/^−^ mouse models further confirms that omentin-1 overexpression reduces plasma total cholesterol levels and atherosclerosis plaque formation by promoting ABCA1-mediated cholesterol efflux from macrophages (14, 17). Conversely, decreased omentin-1 levels can disrupt lipid balance, promoting hepatic triglyceride synthesis and impeding the normal conversion of VLDL to HDL, thereby inducing dyslipidemia and accelerating atherosclerosis progression (17).

#### 3.1.3 Enhancement of insulin sensitivity

Insulin resistance is a well-established risk factor for atherogenesis and its progression. This insulin-sensitizing adipokine specifically potentiates insulin-mediated glucose uptake in adipocytes without altering basal uptake (9). *In vitro* experiments show that recombinant omentin-1 protein can effectively reverse insulin resistance induced by conditioned medium from T2DM patients’ EAT in rat cardiomyocytes, restoring insulin-mediated Akt phosphorylation (18). Moreover, by attenuating systemic inflammation through inhibition of pro-inflammatory signaling (e.g., by hs-CRP and TNF-α), omentin-1 indirectly improves insulin sensitivity (25). Collectively, low omentin-1 levels signify a metabolically compromised state and likely contribute to coronary artery disease (CAD) pathogenesis by impairing insulin signaling.

#### 3.1.4 Anti-inflammatory and antioxidant effects

Inflammation is a complex and tightly regulated biological response of vascular and immune cells to harmful stimuli such as lipopolysaccharide, metabolic stress, and tissue injury. In CVD, chronic low-grade inflammation is characterized by sustained activation of endothelial cells, macrophages, and vascular smooth muscle cells, leading to persistent production of pro-inflammatory cytokines and reactive oxygen species that promote vascular dysfunction and atherogenesis (19, 21, 22). At the molecular level, these inflammatory responses are mediated by interconnected signaling pathways, including NF-κB, Toll-like receptor 4 (TLR4)/MyD88, and Janus kinase/signal transducer and activator of transcription (JAK/STAT) cascades. Wang et al. demonstrated that SIRT6 protects endothelial cells from lipopolysaccharide-induced inflammation by suppressing NF-κB activation and reducing inflammatory cytokine expression, highlighting the importance of transcriptional regulation in endothelial inflammatory responses (26). Similarly, Bao et al. identified TNK1 as a novel mediator of atherosclerotic inflammation via activation of the Tyk2/STAT1 pathway, further emphasizing that vascular inflammation is governed by tightly controlled kinase-driven signaling networks (27).

Within this inflammatory framework, adipokines such as omentin-1 function as important modulators of vascular immune homeostasis. Omentin-1 exerts anti-inflammatory effects primarily through the regulation of macrophage polarization and suppression of pro-inflammatory signaling cascades. In lipopolysaccharide-stimulated macrophages, omentin-1 attenuates TLR4/MyD88-dependent NF-κB activation and reduces the production of pro-atherogenic cytokines (14, 24). It also inhibits inflammasome activation and mitigates macrophage-mediated inflammatory responses, contributing to plaque stabilization in experimental atherosclerosis models (19, 20).

Beyond macrophages, omentin-1 modulates inflammatory signaling in vascular smooth muscle cells by suppressing oxidative stress–related pathways and downstream inflammatory responses (21). Moreover, circulating omentin-1 levels are inversely correlated with systemic inflammatory markers such as IL-6 and hs-CRP (28, 29), supporting its systemic anti-inflammatory role.

The antioxidant properties of omentin-1 synergize with its anti-inflammatory effects. By reducing intracellular reactive oxygen species accumulation and enhancing cellular antioxidant defense mechanisms, including activation of the Nrf2 pathway (22), omentin-1 contributes to the maintenance of redox homeostasis and attenuation of vascular injury.

Collectively, these findings indicate that omentin-1 attenuates vascular inflammation through coordinated modulation of immune cell polarization, inflammatory signaling networks, and redox balance, thereby contributing to plaque stabilization and slowing atherosclerotic progression.

### 3.2 Vascular endothelial protection axis

Endothelial dysfunction is characterized by reduced bioavailability of endothelium-derived vasodilators such as NO and increased production of vasoconstrictors. It represents a key initiating factor in atherosclerosis and is closely associated with circulating omentin-1 levels (30, 31). This dysfunction can affect the coronary circulation, exacerbating myocardial ischemia in CAD patients and even inducing microvascular myocardial ischemia mediated by coronary constriction. As a key regulator of vascular endothelial function, omentin-1 maintains endothelial homeostasis through multiple coordinated mechanisms.

Vascular repair is also regulated by coordinated growth-factor signaling. Yang et al. demonstrated that the synergistic actions of vascular endothelial growth factor (VEGF) and stromal cell-derived factor-1α (SDF-1α) affect both endothelial progenitor cells (EPCs) and VSMCs, providing complementary evidence that vascular regeneration and remodeling are controlled by multiple interacting cellular pathways (32). These findings broaden the mechanistic context of endothelial repair; however, they should not be interpreted as evidence that omentin-1 directly activates the VEGF/SDF-1α pathway.

Reduced number and impaired function of EPCs are significant markers of vascular endothelial injury, increased CVD risk, and poor prognosis. Omentin-1 improves TNF-α-induced EPC dysfunction by inhibiting the p38 mitogen-activated protein kinase (p38 MAPK)/cAMP response element-binding protein (CREB) signaling pathway. This effect is associated with restored proliferation, improved tube formation, and reduced apoptosis. Moreover, adenovirus-mediated exogenous omentin-1 overexpression in EPCs exhibits a stronger ability to inhibit neointimal hyperplasia after rat carotid artery injury, potentially offering a strategy for treating in-stent restenosis (7).

NO is a cornerstone of endothelial homeostasis. Experimental evidence suggests that omentin-1 may enhance NO bioavailability through several mechanisms. First, it activates the AMPK/peroxisome proliferator-activated receptor δ (PPAR-δ) pathway to restore Akt phosphorylation, thereby selectively inducing endothelial nitric oxide synthase (eNOS) phosphorylation at Ser1177 and mitigating endoplasmic reticulum (ER) stress-induced endothelial damage. Second, in an endothelium-dependent manner, it stimulates NO release, directly antagonizing norepinephrine-induced vasoconstriction to mediate vasodilation. Furthermore, omentin-1 inhibits VSMC osteogenic differentiation through the phosphatidylinositol 3-kinase (PI3K)/Akt pathway, thereby delaying arterial calcification, improving vascular compliance, and ultimately preserving endothelial function (33–35).

Inflammation and endothelial dysfunction mutually reinforce each other, driving vascular pathology. *In vitro* experiments using human umbilical vein endothelial cells (HUVECs) show that omentin-1 exerts protective effects by activating the AMPK/eNOS/NO signaling pathway. In addition, it inhibits JNK activation and reduces TNF-α-induced COX-2 expression, thereby alleviating endothelial cell injury. Furthermore, omentin-1 effectively inhibits free fatty acid-induced NF-κB pathway activation, significantly downregulating the expression of monocyte chemoattractant protein-1 (CCL2), pro-inflammatory cytokines like IL-6 and IL-1β, and intercellular adhesion molecule-1 (ICAM-1), thereby blocking the amplification of the inflammatory cascade (6). These findings support a regulatory role for omentin-1 in the inflammation–endothelial dysfunction axis.

Further research reveals a critical vicious cycle between omentin-1 and vascular endothelial function. In populations with obesity-related metabolic disorders, particularly in T2DM complicated by diabetic foot syndrome (DFS), circulating omentin-1 levels show a significant positive correlation with vascular endothelial function, as assessed by the reactive hyperemia index (RHI). These patients exhibit concurrent reductions in plasma omentin-1 concentrations and more severe endothelial dysfunction, suggesting that obesity-related metabolic disturbances promote both omentin-1 downregulation and endothelial impairment, likely mediated by chronic low-grade inflammation (31). Notably, diminished omentin-1 expression further attenuates its protective effects on endothelial cells, thereby exacerbating endothelial injury and accelerating vascular lesion progression. This self-perpetuating cycle further supports the potential protective role of omentin-1 in endothelial homeostasis. These observations further suggest that exogenous omentin-1 supplementation or targeted modulation of its downstream signaling warrants investigation as a potential strategy to interrupt this pathological cycle; however, clinical efficacy remains to be established.

### 3.3 Cardioprotective axis

Animal studies have shown that omentin-1 attenuates myocardial ischemia-reperfusion injury and suppresses cardiomyocyte apoptosis through AMPK- and Akt-dependent signaling. These effects contribute to significant functional recovery after acute ischemia (36). Experimental intervention studies further demonstrate that adipose tissue-specific omentin-1 overexpression activates the Sirtuin 3 (SIRT3)/Forkhead box O3a (FOXO3a) axis, which attenuates post-infarction injury and retards heart failure progression by preserving mitochondrial homeostasis and promoting mitophagy (37). Additionally, evidence indicates that omentin-1 is significantly associated with the early improvement of myocardial function after infarction. Furthermore, by suppressing cardiac hypertrophy and attenuating ventricular dilation, it mitigates maladaptive post-infarct remodeling, supporting the potential cardioprotective relevance of omentin-1 (38, 39).

In addition to omentin-1-mediated cardioprotective signaling, myocardial remodeling is also influenced by complementary anti-fibrotic and cellular-aging pathways. Calcitonin gene-related peptide (CGRP) has been reported to inhibit cardiac-fibroblast senescence and attenuate cardiac fibrosis through upregulation of Klotho expression (40), providing a complementary example of how modulation of cellular senescence may influence adverse myocardial remodeling. However, this evidence should be regarded as complementary rather than omentin-1-specific, because a direct regulatory relationship between omentin-1 and the CGRP/Klotho pathway has not been established.

Similarly, emerging evidence indicates that G protein–coupled estrogen receptor (GPER) plays an important regulatory role in cardiovascular physiology and pathology, modulating inflammation, vascular tone, and myocardial remodeling (41). These observations provide a broader mechanistic context for understanding cardioprotective signaling. However, whether omentin-1 directly modulates GPER activity, acts downstream of GPER-related signaling, or functions through an independent pathway remains unknown and requires further investigation.

Beyond its role in injury repair, omentin-1 is also crucial for normal cardiac development and metabolic maturation. Yang et al. demonstrated that omentin-1 directly binds to bone morphogenetic protein 7 (BMP7), competitively inhibiting its interaction with the activin type IIB receptor (ActRIIB). This binding suppresses the activation and nuclear translocation of downstream signaling molecules, including SMAD1, Yes-associated protein (YAP), and p38 MAPK. As a result, omentin-1 induces cardiomyocyte cell cycle arrest and promotes metabolic maturation, which is characterized by an enhanced reliance on fatty acid oxidation. This mechanism is conserved in both mouse and human cardiomyocytes. Omentin-1 deletion delays myocardial maturation in young mice, leading to cardiac enlargement and dysfunction in adulthood (42). This finding underscores the pivotal role of omentin-1 in establishing the metabolic foundation for cardiac homeostasis and preventing developmental-origin cardiac dysfunction.

Further *in vitro* evidence demonstrates that conditioned medium from the EAT of T2DM patients impairs contractile function in rat cardiomyocytes, characterized by reduced sarcomere shortening, decreased contraction velocity, and aberrant calcium handling (e.g., diminished peak Ca^2+^ fluorescence and delayed decay). These deficits in calcium transients and contractility are completely reversed and partially restored, respectively, by recombinant omentin-1, supporting a role for omentin-1 in modulating calcium handling and cardiomyocyte contractile function (18).

Moreover, omentin-1 suppresses pathological myocardial hypertrophy by inhibiting the activation of the TLR4/MyD88/NF-κB signaling pathway and subsequent phosphorylation of extracellular signal-regulated kinase (ERK), supporting a potential role for omentin-1 in limiting pathological ventricular remodeling (43).

The cardiovascular protective mechanisms of omentin-1 and relevant therapeutic prospects outlined above are illustrated in **Figure 1**.

**Figure 1:**
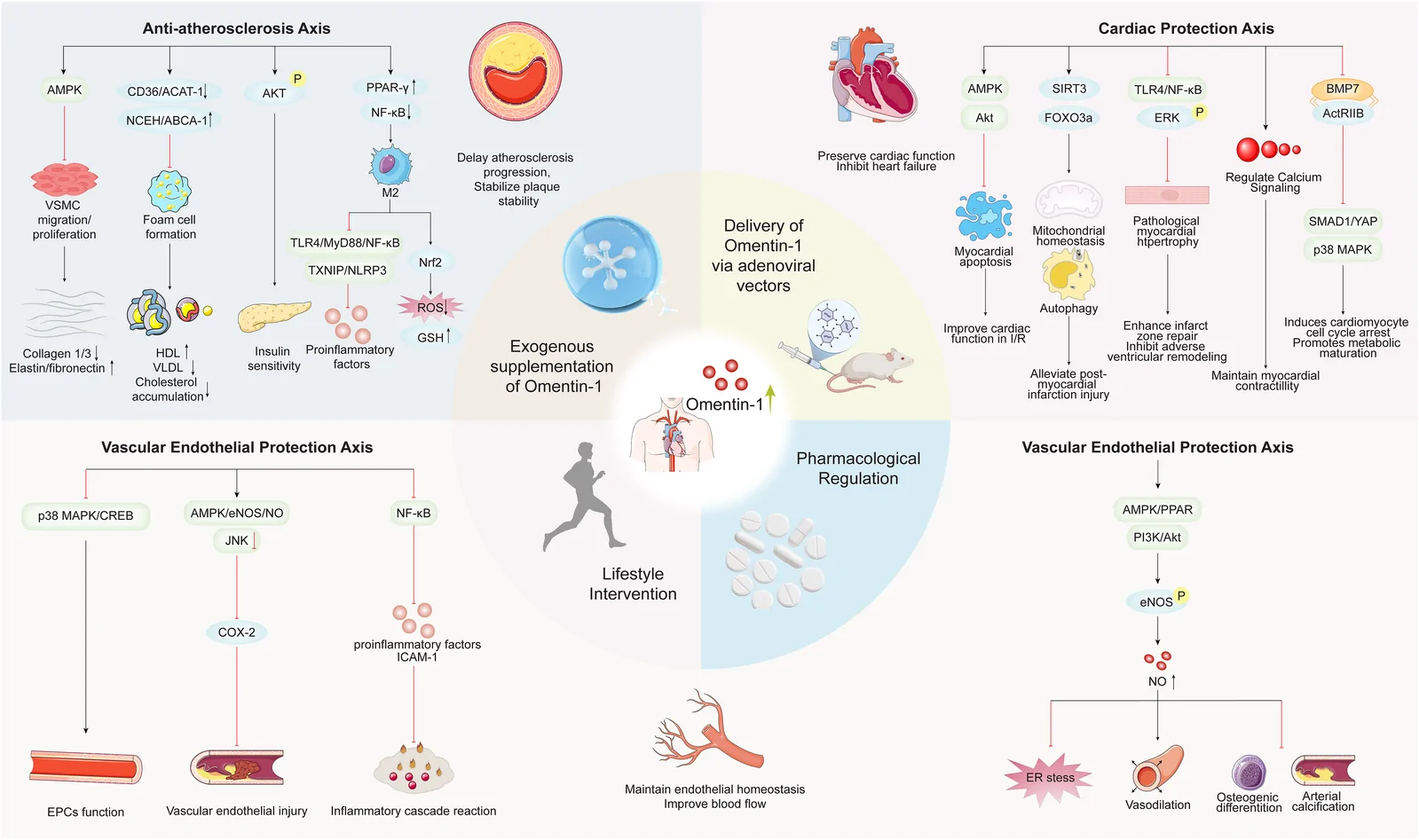
Cardiovascular protective mechanisms of omentin-1. Omentin-1 exerts cardiovascular protective effects via multi-axis and multi-target regulation. This schematic summarizes three functional axes: the anti-atherosclerosis axis, cardioprotective axis, and vascular endothelial protection axis, which jointly mediate its inhibitory effects on vascular injury, inflammation, oxidative stress, pathological myocardial hypertrophy and adverse vascular remodeling. Based on these biological effects, potential translational strategies, including exogenous supplementation, adenovirus-mediated delivery, lifestyle intervention and pharmacological modulation, are also outlined in the diagram.

## 4 Omentin-1 as a novel biomarker and therapeutic target in cardiovascular diseases: from screening to prognosis

Accumulating evidence suggests that circulating omentin-1 may have potential as a biomarker in CVD. Patients with stable CAD exhibit significantly lower circulating omentin-1 levels, which appear to be independent of conventional risk factors such as obesity, hypertension, and dyslipidemia. This association suggests that circulating omentin-1 may reflect aspects of the underlying CAD pathology and may have potential value as a candidate biomarker (44, 45). The reliability of a candidate biomarker also depends on its stability; omentin-1 has shown high intra-individual stability over a 6-month period (ICC = 0.81), supporting further evaluation of its potential utility in clinical risk assessment (16). Nevertheless, its clinical utility for screening and risk stratification remains to be validated in larger prospective cohorts.

Importantly, the clinical interpretation of omentin-1 should take the hierarchy of available evidence into account. Mechanistic effects have largely been established in cellular and animal models, whereas most human evidence remains observational, with limited interventional clinical data. Therefore, associations between circulating omentin-1 and cardiovascular phenotypes should not be interpreted as proof of causality, and its proposed roles as a biomarker or therapeutic target should currently be regarded as investigational rather than clinically validated.

### 4.1 Omentin-1 in coronary artery disease: stage-specific expression patterns and prognostic significance

Omentin-1 expression demonstrates marked tissue specificity and dynamic alterations throughout the pathological stages of CAD. Importantly, circulating omentin-1 levels in CAD are not uniformly inverse and appear to vary according to disease stage. This complexity opens new avenues for research aimed at refining CAD evaluation.

#### 4.1.1 Omentin-1 expression in chronic coronary disease

In patients with chronic coronary disease (CCD), a stable stage of CAD, omentin-1 expression is consistently downregulated. Multiple studies have consistently demonstrated significantly reduced levels of omentin-1, both in circulation and derived from EAT, in CCD patients compared to non-CAD individuals. Furthermore, its expression is even more markedly decreased within EAT adjacent to stenotic coronary segments (44, 46). This phenomenon reflects a core feature of the pathological state in CCD. First, it directly indicates underlying metabolic disorders, such as obesity and insulin resistance, as well as persistent vascular injury (e.g., intimal thickening and matrix remodeling). More importantly, it reflects impaired synthetic function of the major sources of omentin-1, namely EAT and VAT. These metabolic abnormalities are negatively associated with circulating omentin-1 levels and may further exacerbate its reduction (47, 48). Given the close association between omentin-1 levels and the dual pathology of metabolic abnormality and vascular injury, it may serve as a potential precision biomarker for CCD. Its key clinical value lies in assisting traditional risk factors (e.g., blood pressure, blood lipids) in achieving precise risk stratification of CCD patients—particularly those with comorbid metabolic abnormalities—thereby enabling a more objective assessment of the potential severity of the disease.

#### 4.1.2 Omentin-1 expression levels in acute coronary syndrome

In contrast to the relatively stable pattern observed in CCD, omentin-1 expression in ACS appears dynamic, and current findings on circulating levels remain inconsistent, with studies reporting both increases and decreases. This apparent discrepancy may reflect a combination of biological heterogeneity, differences in tissue compartment and disease stage, and methodological variation among studies.

On one hand, elevated circulating omentin-1 may represent a compensatory response to acute ischemic stress. Specific investigations have revealed that compared to hypertensive controls without ACS, patients with ACS exhibit significantly lower omentin-1 levels in the coronary endothelium and EAT. In contrast, relative elevations are observed within macrophages/smooth muscle cells of atherosclerotic plaques and in the circulation (14). This downregulation in EAT is potentially associated with the epithelial-mesenchymal transition process in this tissue (49). During the acute ischemic phase of ST-segment elevation myocardial infarction (STEMI), serum omentin-1 levels peak at hospital admission and subsequently decline over the following 72 hours, a temporal pattern that has been interpreted as potentially consistent with an acute compensatory response (38). Furthermore, among patients with severe coronary artery occlusion (≥90%), plasma omentin-1 levels increase with the degree of stenosis and independently correlate with better coronary collateral circulation formation (50). Collectively, these observations are compatible with the hypothesis that increased circulating omentin-1 during acute ischemia may represent a compensatory response; however, they do not establish that such elevations directly limit myocardial injury or promote repair.

On the other hand, reduced circulating omentin-1 levels may reflect impaired adipose-tissue production or broader metabolic dysfunction. Such a pattern has been reported in non-ST-segment elevation ACS, including NSTEMI and unstable angina (UA), although the mechanisms underlying these reductions remain uncertain. A case-control study demonstrated that serum omentin-1 levels were lower in both ACS and stable angina pectoris (SAP) patients compared to healthy controls, with the ACS group exhibiting the lowest levels (45). Corroborating this, the KOZANI study reported significantly lower circulating omentin-1 levels in AMI patients upon admission than in healthy individuals, which subsequently increased significantly after a 6-month follow-up; the authors proposed that suppression of systemic inflammation might be key to this late increase (51).

Importantly, circulating and tissue omentin-1 measurements should not be assumed to represent the same biological compartment. Reduced omentin-1 expression in EAT or coronary vascular tissue may coexist with relatively elevated circulating concentrations, potentially reflecting differences in local production, systemic release, or compensatory responses during acute ischemic stress. Conversely, lower circulating concentrations may be observed in the presence of chronic metabolic dysfunction or impaired adipose-tissue function. Therefore, apparently conflicting findings in ACS may reflect differences in tissue compartment, disease phenotype (e.g., STEMI versus NSTEMI/UA), disease stage and ischemic duration, sampling time relative to symptom onset or reperfusion, inflammatory and metabolic status, renal function, concomitant medications, and other patient characteristics. Methodological factors, including assay platform, antibody specificity, sample type and processing, and study design, may further contribute to between-study heterogeneity.

Accordingly, altered omentin-1 levels in ACS cannot currently be interpreted as evidence of a single biological process. Depending on the clinical context, they may represent a compensatory response to acute injury, reflect underlying metabolic or tissue dysfunction, or serve as a marker of disease burden or severity. Current observational evidence does not establish whether these changes have a causal role in ACS pathophysiology.

Taken together, the heterogeneity of omentin-1 findings in ACS is likely multifactorial rather than attributable solely to ACS subtype or sampling time. Future studies should therefore incorporate serial measurements across well-defined ACS phenotypes, standardized sampling and assay procedures, and detailed characterization of metabolic status, renal function, concomitant medications, and other relevant clinical variables. Such longitudinal studies are needed to determine whether dynamic changes in omentin-1 provide independent prognostic information and, more importantly, whether they reflect causal involvement, compensatory regulation, or disease severity.

#### 4.1.3 The association between circulating Omentin-1 levels and prognosis

Circulating omentin-1 levels are associated with the risk of major adverse cardiovascular events (MACE) in different populations. However, current findings remain heterogeneous. This association appears to depend strongly on the population studied. The controversy may reflect the divergent roles of omentin-1 under different physiological and pathological conditions. This complexity may also provide a useful perspective for individualized prognostic assessment.

In populations with metabolic abnormalities or organ damage, decreased omentin-1 levels are significantly associated with an adverse prognosis. Results from a cross-sectional study showed that reduced omentin-1 levels were associated with cardiovascular dysfunction in patients with T2DM, including greater left ventricular diastolic dysfunction, reduced vascular elasticity, and increased circulatory load (18). Research focusing on T2DM patients with peripheral artery disease (PAD)/critical limb-threatening ischemia (CLTI) further showed that low baseline omentin-1 levels were significantly associated with an increased risk of MACE within 12 months after lower extremity revascularization (LER) (52). Another prospective study focusing on diabetic patients undergoing hemodialysis showed that decreased serum omentin-1 levels were independently associated with an increased risk of all-cause mortality (53). In these populations, low omentin-1 levels may reflect a state in which metabolic inflammation and endothelial injury are more pronounced, although a direct pathogenic role has not been established.

Conversely, studies involving healthy individuals or patients with stable coronary artery disease (CAD) confirmed by coronary angiography yield opposing conclusions. Several prospective studies have indicated that higher circulating omentin-1 levels are positively associated with an increased risk of cardiovascular events (13, 54, 55). Similarly, a prospective study with a follow-up period of up to 14 years, analyzing diabetic patients, demonstrated that elevated serum omentin-1 levels were significantly associated with an increased risk of primary cardiovascular events, stroke, and cardiovascular mortality, independent of other common cardiovascular risk factors (56). A potential explanation is that in these patients, elevated omentin-1 may not be a direct risk factor but rather a compensatory counter-regulatory response triggered by perceived high underlying risk. However, whether such elevations represent an effective protective response or simply reflect greater underlying disease burden remains uncertain.

Taken together, the available clinical evidence does not establish whether altered circulating omentin-1 is a causal determinant of cardiovascular outcomes, an endogenous compensatory response to vascular or myocardial injury, a consequence of impaired adipose-tissue function, or a biomarker reflecting the severity of an underlying metabolic-inflammatory state. These possibilities are not mutually exclusive and may vary across populations, cardiovascular phenotypes, and disease stages. Longitudinal studies incorporating repeated omentin-1 measurements, causal-inference approaches, and mechanistically informative human investigations will be required to distinguish causality from reverse causation and compensatory regulation.

The possible reasons for the discrepancies between circulating omentin-1 levels and patient prognosis are as follows: (1) Differences in study populations and their baseline characteristics (e.g., age, sex, ethnicity, geographic distribution, underlying treatment status, such as statin use); (2) Variations in study design and detection methods (e.g., sample size, sensitivity and specificity of omentin-1 detection kits, standardization of sample collection, timing of measurement relative to MI); (3) Complexity of causality and regulatory mechanisms – cross-sectional studies cannot determine the temporal sequence between omentin-1 and CAD, and omentin-1’s effects might be modulated by metabolic status (e.g., adiponectin levels, MetS comorbidity), involving complex molecular interactions leading to heterogeneous effects under different pathological conditions.

Regarding patients with acute cardiovascular events, the direct association between omentin-1 and prognosis remains to be further elucidated. This uncertainty primarily stems from the bidirectional controversy of “compensatory upregulation versus impaired synthesis” of omentin-1 during an ACS. Furthermore, there is a notable lack of dynamic investigations stratified by specific ACS subtypes. Future studies should incorporate longitudinal monitoring at multiple time points across different patient subtypes to systematically examine the association of its dynamic changes, from the acute phase to the convalescent period, with post-infarction cardiac function and the risk of recurrent MACE, thereby addressing this critical gap in prognostic assessment.

Detailed clinical evidence regarding omentin-1 expression patterns and prognostic significance across different CAD stages is summarized in **Table 1**.

**Table 1:** The role of Omentin-1 in CAD clinical studies.

Study category	Disease Type	Reference		Sample	Circulating Omentin-1 Levels	Outcomes	Discussion
Association between Acute and Chronic Ischemic States of Different Coronary Artery Disease (CAD) and Prognosis	Chronic Ischemic: (e.g.,CAD)Elbow	Shibata et al. (2011) (44)		*N* = 139 -78 CAD -61 healthy males	↓	Circulating Omentin-1 levels in CAD patients were significantly lower than those in healthy individuals	Circulating Omentin-1 levels are significantly reduced in patients with chronic CAD. This reduction reflects both underlying metabolic dysregulation and persistent tissue injury, as well as impaired Omentin-1 synthesis in EAT. Consequently, Omentin-1 represents a promising biomarker for risk stratification of CAD and related metabolic cardiovascular risk.
		Yu Du et al. (2016) (46)		*N* = 40 -28 coronary heart disease (CAD) group -12 non-coronary heart disease (NCAD) group	↓	Both circulating Omentin-1 levels and EAT-derived Omentin-1 levels in CAD patients were significantly lower than those in non-CAD individuals	
	Acute Ischemic: (e.g.,ACS)Elbow	Viewpoint 1:↑	Watanabe et al. (2016) (14)	*N* = 111 -69 ACS -21 non-ACS -21 healthy volunteers	↑	Circulating Omentin-1 levels in ACS patients were significantly higher than those in non-ACS patients and healthy individuals	Current evidence on circulating Omentin-1 levels in acute ischemia (e.g., ACS) is conflicting. Reports describe either elevated levels, potentially indicating a compensatory protective response (e.g., promoting collateral circulation or mitigating ischemic injury), or decreased levels, possibly due to acute ischemia-induced impairment in synthesis/release. Resolving this discrepancy requires future large-scale, multicenter, longitudinal studies that enroll diverse ACS subtypes (e.g., STEMI, NSTEMI, UA). These studies should track Omentin-1 dynamics from the acute phase through recovery and correlate these changes with infarct size, degree of cardiac dysfunction, and long-term outcomes. This will clarify Omentin-1's pathophysiological role in acute coronary events and its utility as a biomarker for assessing severity and prognosis
			Saddic et al. (2017) (49)	*N* = 140 -Patients undergoing elective aortic valve replacement (acute ischemia)	↑	After acute myocardial ischemia, Omentin-1 mRNA expression in left ventricular tissue was significantly decreased, while circulating plasma Omentin-1 levels were significantly increased	
			Zhu et al. (2019) (38)	*N* = 52 -STEMI	↑	Circulating Omentin-1 levels peaked at admission in STEMI patients and decreased significantly within 72 h	
			Zhou et al. (2017) (50)	*N* = 142 -Patients with ≥90% diameter stenosis in at least one major epicardial coronary artery confirmed by CAG	↑	Circulating Omentin-1 levels increased with the aggravation of severe stenosis (>90%) and were positively correlated with collateral circulation	
		Viewpoint 2:↓	Zhong X, et al. (2011) (45)	*N* = 207 – 127 with ACS (61 AMI, 66 UAP) – 28 with SAP – 52 healthy subjects	↓	Circulating Omentin-1 levels in the ACS group were lower than those in the healthy group and the SAP group	
			Kadoglou et al. (2015) (51)	*N* = 110 – 78 with AMI – 32 healthy subjects	↓	Omentin-1 levels at AMI admission were lower than those in healthy individuals; after 6 months of follow-up, levels were higher than baseline	
	Association with Prognosis of Adverse Cardiovascular EventsElbow	Negative CorrelationElbow	Greulich S et al. (2013) (18)	*N* = 92 – 78 patients with type 2 diabetes mellitus (T2DM) – 14 healthy subjects	–	Decreased Omentin-1 levels in T2DM patients exacerbate left ventricular diastolic dysfunction, reduce vascular elasticity, and increase circulatory load, thereby further promoting cardiovascular dysfunction.	The association between circulating omentin-1 levels and prognosis remains controversial. Some studies suggest that low omentin-1 is a potential pathogenic factor, negatively correlating with adverse cardiovascular risk. Paradoxically, several prospective studies (e.g., by Saely et al. and Menzel et al.) report that high omentin-1 levels increase the risk of cardiovascular events, including stroke. This positive association was particularly prominent in metabolically healthy individuals in the Menzel et al. study, leading to the hypothesis that omentin-1 may not be a direct risk factor but rather a compensatory counter-regulatory response to high underlying cardiovascular risk, albeit insufficient to offset it. Future large-scale, long-term follow-up studies are needed to clarify this relationship across different populations and metabolic subgroups to optimize risk stratification and precision management.
			Biscetti et al. (2020) (52)	*N* = 207 – All with T2DM complicated by peripheral artery disease/chronic limb-threatening ischemia (PAD/CLTI) and undergoing lower extremity revascularization (LER)	–	Baseline low Omentin-1 levels were significantly associated with an increased risk of major adverse cardiovascular events (MACE) after LER in T2DM patients with PAD/CLTI.	
			Kocijancic et al. (2015) (53)	N=120 – Patients with diabetic hemodialysis (HD)	–	Decreased serum Omentin-1 levels are an independent risk factor for all-cause mortality in diabetic HD patients.	
			Narumi et al. (2014) (57)	*N* = 156 – 130 patients with heart failure (HF) – 20 subjects without obvious heart disease	–	Decreased serum Omentin-1 levels were significantly associated with adverse cardiac prognosis in HF patients.	
		Positive CorrelationElbow	Saely et al. (2016) (54)	*N* = 295 – 161 patients with coronary artery disease (CAD) – 134 subjects without significant CAD	–	Among patients undergoing coronary angiography (CAG), higher circulating Omentin-1 levels were significantly associated with a higher risk of cardiovascular events.	
			Menzel et al. (2016) (55)	*N* = 2084 – 400 subjects with incident CVD(including myocardial infarction [MI] and stroke) – 1684 non-case subjects	–	Circulating Omentin-1 levels were positively correlated with stroke risk, and this association was stronger in metabolically healthy populations.	
			Niersmann et al. (2022) (56)	*N* = 933 – Patients with T2DM	–	In T2DM patients, higher serum Omentin-1 concentrations were significantly associated with an increased risk of major cardiovascular events.	

↑ indicates increased measured circulating Omentin1 concentrations.

↓ indicates decreased measured circulating Omentin1 concentrations.

### 4.2 Omentin-1 and heart failure

Studies show that serum omentin-1 levels are lower in HF patients compared to those without heart disease. A single-center study with a median follow-up of 399 ± 378 days showed that decreased serum omentin-1 levels were significantly associated with an increased risk of adverse cardiac outcomes in HF patients, suggesting serum omentin-1 as a potential prognostic marker for risk stratification in HF (57). Another observational study found that circulating omentin-1 levels were significantly lower in patients with heart failure with preserved ejection fraction (HFpEF) than in controls. The HFpEF group also had significantly higher left ventricular diastolic function indices (E/e’ ratio). Higher omentin-1 levels were associated with a lower risk of HFpEF, suggesting that omentin-1 may have potential relevance to HFpEF risk. ROC analysis showed that the diagnostic efficacy of omentin-1 for HFpEF was comparable to N-terminal pro-brain natriuretic peptide (NT-proBNP), with a higher AUC observed for omentin-1 than for NT-proBNP (AUC = 0.809 vs. 0.674) in the 70–80 age group. However, this finding requires validation in independent cohorts. Furthermore, high circulating omentin-1 levels at discharge in acute heart failure patients were associated with reduced long-term weight gain and improved prognosis, suggesting omentin-1 as a potential biomarker for disease progression in elderly HFpEF patients (58).

Dilated cardiomyopathy (DCM) is a common cause of HF and sudden cardiac death. Huang et al. reported that plasma omentin-1 levels were significantly lower in DCM patients with overt HF compared to healthy individuals and positively correlated with left ventricular ejection fraction (LVEF); circulating omentin-1 levels were low across different NYHA functional classes in DCM patients. Importantly, lower circulating omentin-1 levels were independently associated with the presence of DCM before NT-proBNP was added to the regression model; however, this association was no longer significant after NT-proBNP was included (59).

A clinical study showed that serum omentin-1 concentration was negatively correlated with the presence of atrial fibrillation (AF) and atrial remodeling, with progressively lower levels observed from paroxysmal to persistent and permanent AF, suggesting omentin-1’s potential as a biomarker for assessing AF status and atrial remodeling (60). However, this study had a small sample size and was cross-sectional, requiring validation by more longitudinal studies. In summary, omentin-1 levels are closely related to cardiac functional status, and its specific mechanisms in HF and potential as a therapeutic target warrant in-depth investigation.

### 4.3 Omentin-1 and hypertension, MetS, and pulmonary arterial hypertension

Hypertension (HTN) is a central risk factor for CVD morbidity and mortality, and together with MetS, constitutes a major global health burden, significantly increasing the risk of cardiovascular events and all-cause mortality (61–63). Studies indicate that serum omentin-1 levels are significantly lower in HTN patients compared to normotensive individuals, and further decreased in grade 2 hypertension compared to grade 1 (64). Ismail et al. further reported that circulating omentin-1 levels significantly decrease with increasing body weight, HTN progression, and worsening HTN nephropathy (especially stages III-IV), suggesting its potential as a marker reflecting disease severity (29). Studies have shown that plasma omentin-1 levels are significantly lower in MetS patients compared to healthy controls; more crucially, MetS patients who have concomitant carotid atherosclerosis exhibit even lower plasma omentin-1 levels than those without this complication (65). Similarly, a previous study in a specific population (adolescents in Northeast China) also showed that elevated circulating omentin-1 levels were significantly associated with a decreased risk of MetS and obesity, and these inverse associations remained significant after adjustment for relevant covariates (66), further supporting the close link between omentin-1 and metabolic diseases. Given the high prevalence of MetS in HTN patients and its strong association with organ damage and increased cardiovascular risk, the potential value of low omentin-1 levels for identifying high-risk individuals warrants further prospective investigation (2).

Pulmonary arterial hypertension (PAH) is characterized by progressively elevated pulmonary vascular resistance, ultimately leading to right heart failure, poor prognosis, and high clinical mortality (67–70). Its key pathological mechanisms include hypoxia-induced pulmonary arterial endothelial dysfunction, abnormal pulmonary artery contraction/relaxation, excessive proliferation of pulmonary VSMCs, and activation of inflammation and oxidative stress. Basic studies in rat models have shown that omentin-1 upregulates p-AMPKα levels and inhibits endoplasmic reticulum stress through activation of the AMPKα signaling pathway. It also restores eNOS phosphorylation at Ser1177 and promotes NO production. These effects significantly improve hypoxia-induced pulmonary arterial endothelial dysfunction and attenuate pulmonary vascular remodeling and right ventricular hypertrophy (71). These findings provide mechanistic evidence and a potential preclinical rationale for further investigation in PAH. However, these findings are currently limited to animal models, and their applicability to human PAH pathophysiology requires further validation. A critical research gap remains: no study has systematically investigated the dynamic change characteristics of circulating omentin-1 levels in patients with PAH or provided clinical evidence linking these levels to patient prognosis (e.g., disease severity, long-term survival rate). This gap somewhat hinders the clinical translation of omentin-1.

Building upon the current research, future studies should aim to further elucidate the mechanisms of omentin-1 in diseases such as CAD, HF, HTN, and PAH. The scope of this research should then be extended to other cardiovascular diseases, including cardiomyopathies and arrhythmias, to explore its divergent roles and mechanisms across different CVDs and to better clarify potential causal relationships. These efforts will provide a scientific basis for the precise prevention and control of these conditions.

Clinical studies focusing on omentin-1 in non-coronary cardiovascular diseases are summarized in **Table 2**.

**Table 2:** The role of Omentin-1 in clinical studies of cardiovascular diseases beyond coronary artery disease.

Disease Type	Reference	Sample	Circulating Omentin-1 Levels	Outcomes	Discussion
Heart Failure (HF)	Narumi et al. (2014) (57)	N=156 – 136 with HF – 20 subjects without obvious signs of heart disease	↓	Circulating Omentin-1 levels in HF patients were lower than those in individuals without obvious heart disease.	Circulating Omentin-1 levels may serve as a potential prognostic biomarker in HF.
	Su et al. (2024) (58)	N=217 – 115 with heart failure with preserved ejection fraction (HFpEF) – 102 without heart failure	↓	Decreased circulating Omentin-1 was negatively correlated with diastolic dysfunction (E/e'↑) and inflammation; Diagnostic efficacy: Comparable to NT-proBNP overall; in patients aged 70–80 years, omentin-1 showed a higher AUC than NT-proBNP (0.809 vs. 0.674).	
Dilated Cardiomyopathy (DCM)	Huang et al. (2016) (59)	N=145 – 100 with DCM – 45 healthy subjects	↓	Circulating Omentin-1 levels in DCM patients were significantly lower than those in healthy individuals.	Decreased circulating Omentin-1 was positively correlated with LVEF and was independently associated with DCM before NT-proBNP was included in the model, but this association was no longer significant after NT-proBNP inclusion.
Atrial Fibrillation (AF)	Tao et al. (2016) (60)	N=335 – 220 AF group (70 paroxysmal AF, 78 persistent AF, 72 permanent AF) – 115 healthy subjects	↓	Circulating Omentin-1 concentration gradually decreased across AF types (paroxysmal → persistent → permanent).	Serum Omentin-1 may have potential as a biomarker for assessing AF status and atrial remodeling.
Hypertension(HTN)&Metabolic Syndrome(MetS)	Mustafa Çelik et al.(2021) (64)	N=121 – 61 HTN patients (31 Grade 1, 30 Grade 2) – 60 healthy subjects	↓	Serum Omentin-1 levels in HTN patients were lower than those in healthy normotensive individuals; levels in Grade 2 HTN patients were further decreased compared to Grade 1.	Serum Omentin-1 levels are significantly negatively correlated with disease progression, metabolic abnormalities, and the risk of target organ damage in hypertensive patients.
	Ismail et al. (2024) (29)	N=126 – 42 hypertensive patients – 42 hypertensive patients with renal disease – 21 obese subjects – 21 healthy subjects	↓	Circulating Omentin-1 levels in hypertensive patients were significantly lower than those in healthy individuals.	
	Liu et al.(2011) (65)	N=90 – 30 MetS+ atherosclerosis – 30 MetS- atherosclerosis – 30 healthy subjects	↓	Plasma Omentin-1 levels were significantly reduced in patients with MetS and were further decreased in MetS patients with comorbid AS.	
	Cetin Sanlialp et al. (2022) (2)	N=110 – 66 HTN + MetS – 44 HTN	↓	Serum Omentin-1 levels in HTN patients with MetS (median: 46.35 ng/ml) were lower than those in HTN patients without MetS (median: 130.95 ng/ml).	
	Sun et al. (2022) (66)	N=742 – Natural population without specific underlying diseases	↑	Elevated circulating Omentin-1 levels were significantly associated with reduced risks of MetS and obesity; these associations remained significant after adjustment in adolescents from Northeast China.	

↑ indicates increased measured circulating Omentin1 concentrations.

↓ indicates decreased measured circulating Omentin1 concentrations.

### 4.4 Therapeutic potential

Although experimental findings provide a strong biological rationale for targeting omentin-1-related pathways, the therapeutic evidence remains predominantly preclinical. The safety and efficacy of omentin-1-based therapeutic strategies have not yet been established in human cardiovascular disease. The following therapeutic concepts should therefore be interpreted as translational hypotheses and future research directions rather than clinically validated treatment strategies.

#### 4.4.1 Direct therapeutic strategies

Omentin-1 represents a potential therapeutic target based primarily on preclinical evidence. Exogenous administration of recombinant omentin-1 has been shown to attenuate myocardial ischemic injury in experimental animal models through AMPK- and Akt-dependent mechanisms (36). Similarly, adenovirus-mediated elevation of omentin-1 has demonstrated cardioprotective effects in experimental ischemia/reperfusion models (36). However, these findings should not be extrapolated directly to clinical efficacy in humans.

Several important translational issues remain to be addressed. The pharmacokinetic and pharmacodynamic properties of recombinant omentin-1 have not been sufficiently characterized, and key parameters such as circulating half-life, tissue distribution, bioavailability, optimal dose, route and frequency of administration, and duration of biological activity require further investigation. Potential immunogenicity, off-target effects, long-term safety, and interactions with established cardiovascular therapies also require systematic evaluation. Importantly, robust interventional human evidence demonstrating the safety and cardiovascular efficacy of omentin-1-based therapy remains lacking.

Accordingly, recombinant omentin-1 administration, gene delivery, long-acting omentin-1 analogues, and agents targeting omentin-1-related signaling should currently be regarded as investigational strategies. Identification and validation of the functional receptor(s) mediating the cardiovascular effects of omentin-1 will also be important before receptor-directed pharmacological development can be pursued.

#### 4.4.2 Pharmacological modulation of Omentin-1

Emerging evidence indicates that circulating omentin-1 levels can be modulated by commonly used cardiometabolic drugs. Pioglitazone, a peroxisome proliferator-activated receptor gamma (PPARγ) agonist, has been shown to increase omentin-1 levels and improve cardiac function (18). Atorvastatin has also been shown to increase circulating omentin-1 levels in a dose-dependent manner in patients with CAD (72). Metformin treatment has also been associated with increased omentin-1 levels (5, 73). Collectively, these observations indicate that circulating omentin-1 levels may be modulated by several cardiometabolic therapies. However, whether changes in omentin-1 mediate any component of their cardiovascular benefits, merely accompany broader metabolic improvement, or represent an epiphenomenon remains uncertain. Dedicated mechanistic and interventional studies will be required to determine whether omentin-1 contributes causally to the therapeutic effects of these agents.

#### 4.4.3 Combination therapeutic strategies

Given the multifactorial nature of CVD, future studies may investigate whether modulation of omentin-1-related pathways can complement established cardioprotective therapies, including GLP-1 receptor agonists (GLP-1RAs), sodium–glucose cotransporter 2 inhibitors (SGLT2is), angiotensin-converting enzyme inhibitors/angiotensin receptor blockers (ACEIs/ARBs), and beta-blockers. At present, however, direct evidence supporting synergistic cardiovascular effects between these established therapies and omentin-1-targeted interventions is lacking. Such combination approaches should therefore be regarded as hypothesis-generating research directions rather than established therapeutic strategies.

#### 4.4.4 Lifestyle interventions and regenerative strategies

Lifestyle interventions are also closely associated with cardiovascular health. Physical activity has been shown to reduce the prevalence of CVD and cardiovascular mortality risk (74, 75). Studies have shown that interventions such as aerobic exercise (e.g., chronic endurance training) combined with resistance training (76, 77) and caloric restriction (78) can endogenously increase circulating omentin-1 concentrations in humans. Although these interventions have been associated with increased circulating omentin-1 levels, whether omentin-1 contributes causally to their cardiovascular benefits remains uncertain.

In addition, intermittent fasting, particularly when aligned with circadian-rhythm regulation, has been associated with improvements in cardiometabolic health through effects on metabolic reprogramming and inflammatory signaling (79). These findings suggest that feeding time and circadian organization may represent additional variables that should be considered when investigating adipokine biology. However, direct evidence that intermittent fasting produces its cardiovascular effects through modulation of omentin-1 is currently lacking.

Beyond lifestyle modification, regenerative strategies are emerging as a promising therapeutic direction. Stem cell–based therapies have demonstrated potential to restore myocardial function and vascular integrity (80). Whether modulation of omentin-1-related pathways can complement cell-based regenerative strategies remains unknown and warrants experimental investigation.

## 5 Conclusion and future perspectives

Omentin-1 is a pleiotropic adipokine with significant cardiometabolic relevance. Experimental and clinical evidence supports its involvement in modulating atherosclerosis, endothelial dysfunction, myocardial injury, and metabolic homeostasis. Beyond its mechanistic importance, circulating omentin-1 has shown potential as a biomarker for cardiovascular risk stratification and disease monitoring, although its clinical utility remains to be validated.

However, several critical gaps remain. First, the specific receptor(s) mediating omentin-1 signaling and the integration of its downstream pathways require clarification. Second, most clinical data derive from cross-sectional or small-scale studies; robust longitudinal cohorts are necessary to establish causality and define clinically meaningful thresholds. Third, therapeutic translation is constrained by incomplete pharmacokinetic and pharmacodynamic characterization of recombinant omentin-1, including uncertainty regarding its circulating half-life, optimal dosing, and duration of action.

Lifestyle and temporal metabolic factors may represent an additional source of variability in omentin-1 biology. Recent evidence linking intermittent fasting and circadian-rhythm organization with cardiovascular health suggests that future studies evaluating circulating omentin-1 should account for feeding patterns, sampling time, circadian phase, metabolic status, and other lifestyle-related factors (79). However, current evidence does not establish that intermittent fasting or circadian interventions directly alter omentin-1 concentrations or signaling, and this relationship requires dedicated investigation.

Future research should therefore prioritize receptor identification, longitudinal human validation, standardized biomarker assessment, and the development of stable analogs or targeted agonists. Prospective studies should incorporate standardized sampling protocols and carefully account for circadian timing, feeding status, metabolic state, medication use, and other potential confounders when evaluating circulating omentin-1. Early-phase clinical trials assessing safety, pharmacokinetics, pharmacodynamics, and preliminary efficacy in appropriately selected populations will be essential before therapeutic application can be considered. Addressing these gaps will determine whether omentin-1 can progress from an investigational cardiovascular biomarker and preclinical therapeutic candidate toward clinically validated applications.

## Glossary

ABCA1: ATP-binding Cassette Transporter A1
ACAT-1: Acyl-CoA: Cholesterol Acyltransferase 1
ACEIs: Angiotensin-Converting Enzyme Inhibitors
ActRIIB: Activin Type IIB Receptor
ACS: Acute Coronary Syndrome
AF: Atrial Fibrillation
Akt: Protein Kinase B
AMI: Acute Myocardial Infarction
AMPK: AMP-activated Protein Kinase
Ang II: Angiotensin II
ApoE⁻/⁻: Apolipoprotein E Knockout
ARBs: Angiotensin Receptor Blockers.
AUC: Area Under Curve
BMP7: Bone Morphogenetic Protein 7
CAD: Coronary Artery Disease
Ca²⁺: Calcium Ion
CCL2: C-C Motif Chemokine Ligand 2
CCD: Chronic Coronary Disease
CD36: Cluster of Differentiation 36
CLTI: Critical Limb-Threatening Ischemia
COX-2: Cyclooxygenase 2
CREB: cAMP Response Element-Binding Protein
CVD: Cardiovascular Disease
DCM: Dilated Cardiomyopathy
EAT: Epicardial Adipose Tissue
E/e’: Early diastolic mitral inflow velocity/early diastolic mitral annular tissue velocity
eNOS: Endothelial Nitric Oxide Synthase
EPCs: Endothelial Progenitor Cells
ER: Endoplasmic Reticulum
ERK: Extracellular Signal-Regulated Kinase
FOXO3a: Forkhead Box O3a
GLP-1RAs: Glucagon-Like Peptide-1 Receptor Agonists
GPER: G protein–coupled Estrogen Receptor
HDL: High-Density Lipoprotein
HF: Heart Failure
HFpEF: Heart Failure with Preserved Ejection Fraction
hs-CRP: High-Sensitivity C-Reactive Protein
HTN: Hypertension
HUVECs: Human Umbilical Vein Endothelial Cells
ICAM-1: Intercellular Adhesion Molecule-1
ICC: Intraclass Correlation Coefficient
IL-6: Interleukin-6
IL-1β: Interleukin-1β
JAK/STAT: Janus Kinase/Signal Transducer and Activator of Transcription
JNK: c-Jun N-terminal Kinase
LER: Lower Extremity Revascularization
LVEF: Left Ventricular Ejection Fraction
MACE: Major Adverse Cardiovascular Events
MetS: Metabolic Syndrome
MyD88: Myeloid Differentiation Primary Response Gene 88
NCEH: Neutral Cholesterol Ester Hydrolase
NF-κB: Nuclear Factor Kappa-B
NO: Nitric Oxide
Nrf2: Nuclear Factor Erythroid 2-Related Factor 2
NSTEMI: Non-ST-segment Elevation Myocardial Infarction
NT-proBNP: N-terminal pro-Brain Natriuretic Peptide
NYHA: New York Heart Association
oxLDL: Oxidized Low-Density Lipoprotein
PAD: Peripheral Artery Disease
PAH: Pulmonary Arterial Hypertension
p38 MAPK: p38 Mitogen-Activated Protein Kinase
PDGF-BB: Platelet-Derived Growth Factor-BB
PI3K: Phosphatidylinositol 3-Kinase
PPAR-δ: Peroxisome Proliferator Activated Receptor δ
PPARγ: Peroxisome Proliferator Activated Receptor γ
RHI: Reactive Hyperemia Index
ROC: Receiver Operating Characteristic
SAP: Stable Angina Pectoris
SAT: Subcutaneous Adipose Tissue
SGT2is: Sodium–Glucose Cotransporter 2 Inhibitors
SIRT3: Sirtuin 3
SIRT6: Sirtuin 6
SMAD1: Mothers Against Decapentaplegic Homolog 1
SR-A: Scavenger Receptor A
STEMI: ST-segment Elevation Myocardial Infarction
T2DM: Type 2 Diabetes Mellitus
TLR4: Toll-like Receptor 4
TNF-α: Tumor Necrosis Factor-α
TNK1: Tyrosine Kinase Non-Receptor 1
Tyk2: Tyrosine Kinase 2
UA: Unstable Angina
VAT: Visceral Adipose Tissue
VLDL: Very-Low-Density Lipoprotein
VSMCs: Vascular Smooth Muscle Cells
YAP: Yes-Associated Protein
